# Primary Failure Eruption: Genetic Investigation, Diagnosis and Treatment: A Systematic Review

**DOI:** 10.3390/children10111781

**Published:** 2023-11-02

**Authors:** Francesco Inchingolo, Irene Ferrara, Fabio Viapiano, Anna Maria Ciocia, Irene Palumbo, Mariafrancesca Guglielmo, Alessio Danilo Inchingolo, Andrea Palermo, Ioana Roxana Bordea, Angelo Michele Inchingolo, Daniela Di Venere, Gianna Dipalma

**Affiliations:** 1 Department of Interdisciplinary Medicine, School of Medicine, University of Bari “Aldo Moro”, 70124 Bari, Italy; ire.ferra3@gmail.com (I.F.); viapianofabio96@gmail.com (F.V.); anna.ciocia1@gmail.com (A.M.C.); irenepalu@icloud.com (I.P.); m.guglielmo2@studenti.uniba.it (M.G.); ad.inchingolo@libero.it (A.D.I.); angeloinchingolo@gmail.com (A.M.I.); daniela.divenere@uniba.it (D.D.V.); giannadipalma@tiscali.it (G.D.); 2 College of Medicine and Dentistry, Birmingham B4 6BN, UK; andrea.palermo2004@libero.it; 3 Department of Oral Health, Iuliu Hatieganu University of Medicine and Pharmacy, 15 V. Babes Street, 400012 Cluj-Napoca, Romania; roxana.bordea@gmail.com

**Keywords:** primary failure of tooth eruption, PFE, MFE, interceptive orthodontics, pediatric dentistry, pediatric orthodontics, PTHR1, eruption pattern, ankylosis

## Abstract

Aim: The aim of this systematic review is to explore the pathology, diagnosis, treatment, and genetic basis of Primary Failure of Eruption (PFE) in the field of pediatric dentistry and orthodontics. Methods: The Preferred Reporting Items for Systematic Reviews and Meta-Analyses (PRISMA) guidelines were followed for this review. The databases PubMed, Science Direct, Scopus, and Web of Science were searched from 1 July 2013 to 1 July 2023, using keywords “primary failure of tooth eruption” OR “primary failure of eruption” OR “tooth eruption failure” OR “PFE” AND “orthodontics”. The study selection process involved screening articles based on the inclusion and exclusion criteria. Results: A total of 1151 results were obtained from the database search, with 14 papers meeting the inclusion criteria. The review covers various aspects of PFE, including its clinical features, diagnosis, treatment options, and genetic associations with mutations in the PTH1R gene. Differentiation between PFE and Mechanical Failure of Eruption (MFE) is crucial for accurate treatment planning. Orthodontic and surgical interventions, along with multidisciplinary approaches, have been employed to manage PFE cases. Genetic testing for PTH1R mutations plays a significant role in confirming the diagnosis and guiding treatment decisions, although some cases may not be linked to this mutation. Conclusions: This systematic review provides valuable insights into the diagnosis, treatment, and genetic basis of PFE. Early diagnosis and personalized treatment planning are crucial for successful management. Genetic testing for PTH1R mutations aids in accurate diagnosis and may influence treatment decisions. However, further research is needed to explore the complex genetic basis of PFE fully and improve treatment outcomes for affected individuals.

## 1. Introduction

Primary Failure of Eruption (PFE) represents a significant pathology in the field of pediatric dentistry and orthodontics. It is a disorder of dental development in which permanent teeth, despite being fully formed and present below the gum line, do not erupt in the expected time frame or fail to do so at all [1,2].

It is estimated that the incidence of PFE ranges between 0.06% and 1.6% of the general population. It can affect both males and females, although some studies suggest a slight female predominance. It can affect any permanent tooth, but second molars and lower canines are often involved [1,3,4]. Several teeth are more frequently involved and only rarely a single tooth [5].

Timely diagnosis and appropriate treatment of PFE are crucial in preventing further dental complications. A delay in diagnosis may result in more invasive and complex orthodontic treatments in adulthood, such as extractions, orthognathic surgery, or dental implants. A correct diagnosis, in particular, requires an in-depth understanding of the various clinical presentations of PFE, the ability to correctly interpret an orthopantomogram, and comprehension of the appropriate age for permanent tooth eruption [6,7,8].

The exact causes of PFE remain unknown, but several associated risk factors exist. These include genetic anomalies, hormonal disorders, systemic factors like inherited syndromes, and trauma or infections that may affect dental development. In some cases, PFE may be associated with local conditions such as the presence of odontogenic tumours, cysts, or dental space abnormalities [9,10].

The classification of PFE can be based on various parameters. It can be classified as localized or generalized depending on the number of teeth involved. It can also be divided based on the dental arch involved (mandibular or maxillary). In terms of severity, PFE can be mild (eruption delay of less than a year), moderate (delay of 1–2 years), or severe (delay of over 2 years or failed eruption). This classification can help determine the most suitable treatment and predict possible outcomes [11,12,13,14].

Constant research and literature reviews in this field are crucial to improving our diagnostic and treatment strategies [15].

PFE has been linked to mutations in a gene called Parathyroid Hormone 1 Receptor (PTH1R). This gene is responsible for producing a receptor that plays a critical role in controlling bone and dental growth and development.

If a healthcare professional suspects PFE based on a patient’s clinical symptoms, they may suggest genetic testing for mutations in the PTH1R gene. This test usually involves collecting a blood or saliva sample that is then analysed in a specialized laboratory. Genetic testing can help confirm a PFE diagnosis and may also provide important information for treatment planning and genetic counselling [16,17].

Genetic analysis can be an important diagnostic tool in cases of PFE and can be useful in confirming a diagnosis and guiding treatment decisions [10]. However, genetic testing should be considered carefully, taking into account the clinical context and the patient’s symptoms. Currently, as the PTH1R gene is the main gene associated with PFE, genetic testing can identify these mutations, confirming the diagnosis of PFE [18]. However, not all patients with PFE will have mutations in this gene, meaning that a negative test does not rule out the possibility of PFE [3]. Genetic screening tests may be appropriate in cases where PFE is suspected, especially if family history suggests a genetic predisposition. Before undergoing genetic testing, patients should be evaluated by a medical genetics specialist or genetic counsellor. Patients eligible for genetic testing are those with obvious symptoms of PFE, including significant delays in the eruption of permanent teeth, interdental spaces, dental abnormalities, and patients with a family history of PFE [19].

However, it is important to note that not all individuals with PFE will have a mutation in this specific gene, and there are other conditions that can cause symptoms like PFE [20,21]. Therefore, genetic testing should be used as part of a comprehensive diagnostic approach that includes clinical examination and patient medical history [22,23,24].

The PTH1R gene primarily regulates calcium and phosphate levels, crucial for bone and tooth development. It also affects:

1. Renal Function: it regulates calcium and phosphate reabsorption in the kidneys, impacting blood and urine mineral levels [25,26].
2. Endocrine Function: it controls parathyroid hormone (PTH) secretion, influencing calcium and phosphate balance in bones, kidneys, and the intestine [27].
3. Muscle Function: indirectly, it affects muscle contraction by regulating blood calcium levels [28].
4. Nervous System: it indirectly influences nerve function through calcium regulation [29].
5. Cardiovascular Health: proper calcium levels are vital for heart function, influenced by PTH1R [30].
6. Mineral Metabolism: it affects various metabolic processes beyond bones and teeth [31].
7. Immune System: PTH1R may modulate the immune response via calcium signalling [32].

Imbalances in PTH1R can lead to conditions like hyperparathyroidism (high blood calcium) or hypoparathyroidism (low blood calcium), affecting multiple organ systems [33].

In addition to the PTH1R gene, several other genes have been implicated in the development and regulation of PFE. These genes include:

1. Latent TGF-Binding Protein 3 Gene: mutations in this gene have been linked to PFE. This gene is involved in the regulation of transforming growth factor-beta (TGF-β), which plays a role in tooth eruption [34].
2. Collagen Type I Alpha 1 Chain and Collagen Type I Alpha 2 Chain Genes: mutations in these collagen genes can lead to connective tissue disorders, such as Ehlers–Danlos syndrome, which may, in some cases, be associated with PFE [35].
3. Family with Sequence Similarity 20 Member A Gene: mutations in this gene have been linked to amelogenesis imperfecta and gingival fibromatosis syndrome, which can include PFE as a symptom [36].
4. WD Repeat Domain 72 Gene: mutations in this gene are associated with a condition known as Raine syndrome, which can manifest with dental anomalies like PFE [37].

It is important to note that these genetic associations with PFE are still an active area of research, and new genes and mutations may be discovered as our understanding of this condition evolves.

**Figure 1:**
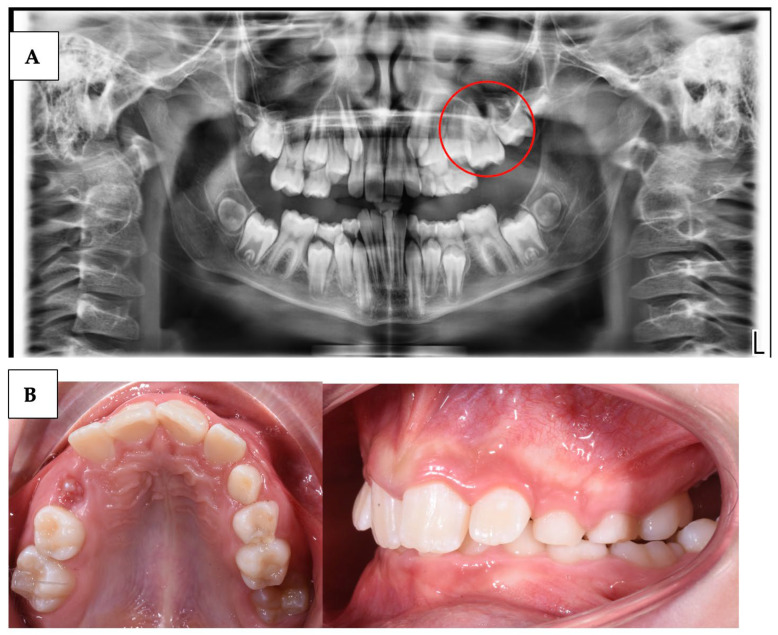
(**A**) Panoramic radiograph of a suspected case of PFE on tooth 26; (**B**) intraoral records of suspected PFE on tooth 26.

The clinical features of PFE can vary depending on the individual patient, but there are some common signs and symptoms:

- Delay in tooth eruption: one of the earliest and most obvious signs of PFE is a delay in the eruption of permanent teeth beyond the expected age (Table 1).
- Excess space between teeth: PFE can result in excessive spacing between teeth due to the absence of the unerupted tooth. This can be more noticeable if multiple teeth are involved.
- Malocclusion and dental misalignment: over time, PFE can lead to malocclusion and dental misalignment issues due to the lack of eruptive force on adjacent teeth.
- Tilted or rotated teeth: in some cases, the teeth adjacent to the site of PFE may tilt or rotate in an attempt to fill the space left by the unerupted tooth.
- Pain or sensitivity: in some cases, PFE can be associated with pain or sensitivity (condition in which a sharp, momentary pain is felt in the teeth when they are exposed to certain thermal, chemical, and mechanical stimuli) at the site of failed eruption, although this is not a common sign.
- Changes on panoramic radiographs: panoramic radiographs may show the fully formed but unerupted tooth, with no apparent physical obstructions to its eruptive path (Figure 1) [3].

**Figure 2:**
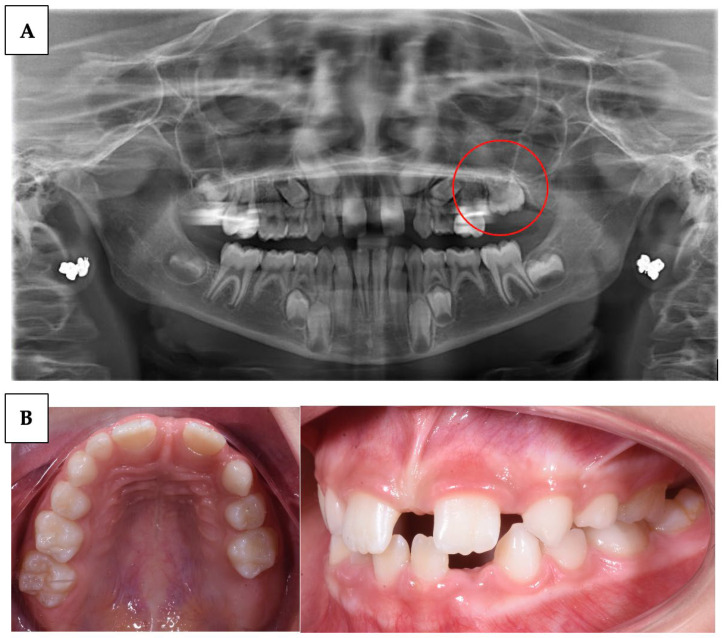
(**A**) Patient with multiple agenesis and suspected case of PFE on tooth 26. (**B**) Intraoral records.

This can lead to a series of long-term orthodontic problems, as improperly erupted teeth can cause displacements, impactions, and occlusal issues (Figure 2) [1,4].

The treatment of PFE varies depending on the severity of the condition, the patient’s age, and the presence of other dental or health issues. In cases where a single tooth is involved, the approach might be to wait and see if the tooth will spontaneously erupt. If it does not erupt within a physiological time frame, orthodontic or surgical intervention may be required. In some cases, treatment may include an orthodontic appliance to create space for the unerupted tooth and facilitate its eruption [5,39]. However, it is important to emphasize that in the case of PFE, due to the intrinsic lack of eruptive force, traditional orthodontic interventions often do not yield stable results. Even after the tooth has been moved into position with orthodontics, it may tend to return to its original position. If the tooth fails to erupt despite orthodontic interventions, or if there is extensive failure of eruption, extraction may be necessary. Subsequently, options may include a bridge, a dental implant, or a removable partial denture to replace the missing tooth.

In some cases, a multidisciplinary approach may be advised, involving a team of specialists, including a pedodontist, an orthodontist, an oral surgeon, and a prosthodontist. Furthermore, if an associated systemic or syndromic condition is present, follow-up by other medical specialists may be necessary. Finally, PFE treatment should be personalized based on the patient’s specific needs.

It’s important to note that PFE can occur either in isolation or as part of broader genetic conditions or syndromes. For example, a higher incidence of PFE has been found in individuals with certain genetic conditions, such as Gardner Syndrome, where the incidence can be over 5% [1,40,41,42].

Numerous dental and oral conditions share clinical manifestations similar PFE, which can lead to confusion in diagnosis [15]. Among these conditions, Ankylosis is characterized by teeth that fuse with the surrounding bone, preventing normal eruption and closely resembling PFE [43]. Impacted teeth, a common occurrence due to various obstructions or space limitations, can also present with non-eruption similar to PFE [44]. Dental crowding, supernumerary teeth (extra teeth), hypodontia (congenital missing teeth), odontogenic cysts, tumours, and retained deciduous (baby) teeth are other conditions that can exhibit clinical symptoms akin to PFE, including failure of tooth eruption [45,46]. Mechanical Failure of Eruption (MFE) is a condition in which a tooth fails to emerge through the gum line due to physical obstructions or other mechanical factors. It can be confused with PFE, as both involve a failure in tooth eruption, but the underlying causes and treatment approaches differ. Accurate diagnosis is crucial for tailoring appropriate treatment strategies for each specific condition [47].

Despite the relatively low incidence in the general population, PFE can have a significant impact on the quality of life of affected patients [48]. It can lead to self-esteem issues related to physical appearance, difficulties in chewing and speaking, and may require prolonged and costly orthodontic treatments [48].

For these reasons, this systematic review investigates PFE as an active research topic in the field of orthodontics and pediatric dentistry.

## 2. Materials and Methods

### 2.1. Protocol and Registration

This systematic review was conducted according to Preferred Reporting Items for Systematic Reviews and Meta-Analyses (PRISMA) and the protocol was registered at PROSPERO under the ID 473137 [49].

### 2.2. Data Sources and Search Strategy

The qualifying criteria were developed using the PICOS (population, intervention, comparison, outcomes, and study design) framework. Pubmed, Science Direct, Scopus, and Web of Science databases were searched from 1 July 2013 to 1 July 2023, using the keywords “primary failure of tooth eruption” OR “primary failure of eruption” OR “tooth eruption failure” OR “PFE” AND “orthodontics”.

### 2.3. Study Selection and Characteristics

This research aims to identify diagnostic criteria and possible treatment strategies for PFE. Articles that met several criteria were included: (1) the study design selected was Randomized Clinical Trials (RCT), case series with more than 3 case reports, clinical trials (CT), retrospective studies (R), or prospective studies (P); (2) participants were human of any age; (3) patient with a diagnosis of PFE; (4) the language selected was English; and (5) only full-text available.

Studies characterized by one of the following exclusion criteria were excluded: (1) the study designs excluded were reviews, letters, comments, case series with less than 3 case reports; and case reports in vitro studies; (2) participants were animal models or dry skulls studies; and (3) articles about case reports with no clear diagnosis of PFE.

#### Quality Assessment

The quality of the included papers was assessed by two reviewers, RF and EI, using ROBINS-I, which is a tool developed to assess the risk of bias in the results of non-randomized studies that compare the health effects of two or more interventions. Seven points were evaluated and each was assigned a degree of bias. A third reviewer (FI) was consulted in the event of a disagreement until an agreement was reached.

### 2.4. Results

**Figure 3:**
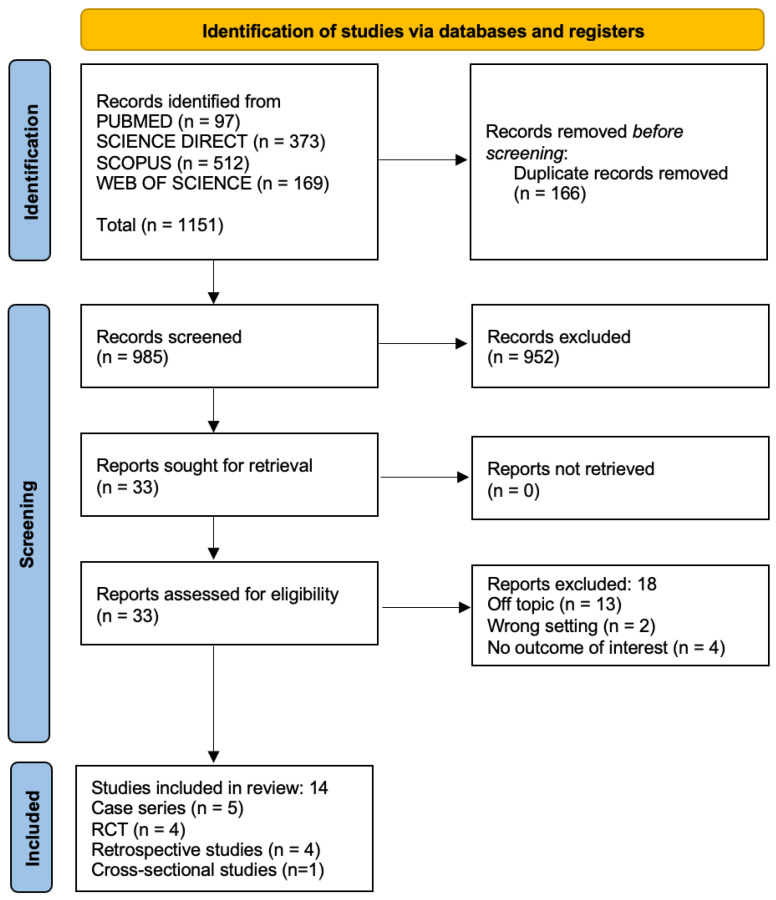
Literature search Preferred Reporting Items for Systematic Reviews and Meta-Analyses (PRISMA) flow diagram.

The electronic database search generated 1151 results. Following duplication elimination, 985 studies were screened for title and abstract. After the abstract screening, 952 papers were rejected, and 33 articles were chosen for the eligibility evaluation. Following the full-text examination, 19 manuscripts were eliminated: 13 off-topic, 2 wrong settings, and 4 with no outcome of interest. Finally, 14 papers were chosen for the systematic review. The selection process and the summary of selected records are shown in Figure 3 and Table 2, respectively.

#### Quality Assessment and Risk of Bias

**Figure 4:**
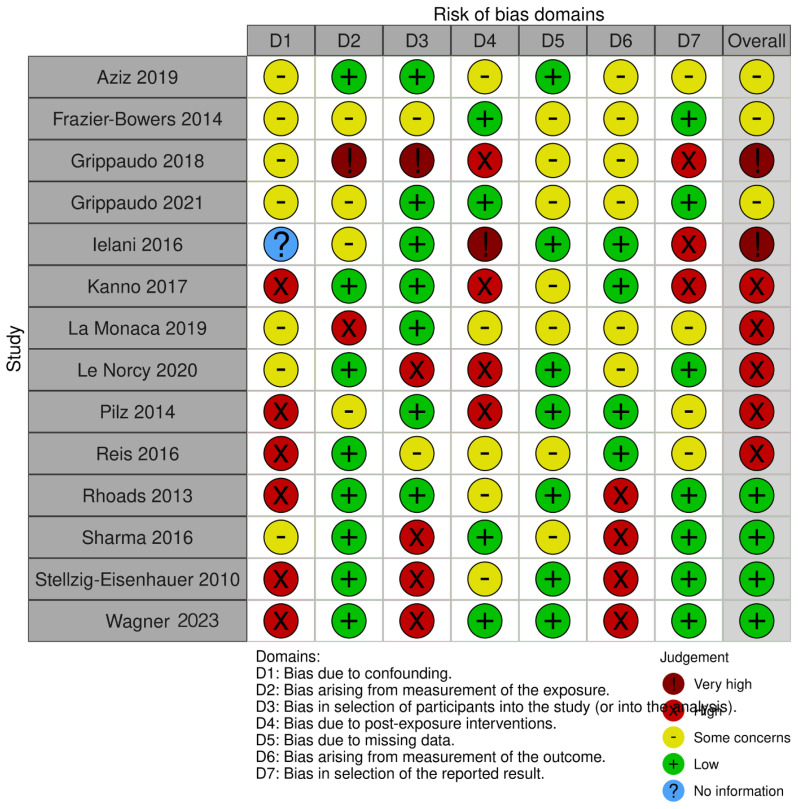
Risk of bias assessment, using Robins Tool [3,10,19,22,50,51,52,53,54,55,56,57,58,59].

The risk of bias in the included studies is reported in Figure 4. Regarding the bias due to confounding, most studies have a high risk. The bias arising from measurement is a parameter with low risk of bias. The majority of studies have low risk of bias due to bias in selection of participants. Bias due to post-exposure cannot be calculated due to high heterogeneity. The bias due to missing data is low in the majority of studies. Bias arising from measurement of the outcome is low. Bias in the selection of the reported results is high in the majority of studies. The final results show that six studies have a low risk of bias, ten studies have a high risk of bias, two have a very high risk of bias, and the remainder have a questionable risk of bias.

## 3. Discussion

### 3.1. PFE Diagnosis

The study conducted by Sharma et al. aimed to differentiate between PFE and MFE of first and second permanent molars and identify successful management strategies for these conditions. PFE is a rare isolated condition causing localized failure of tooth eruption with no other local or systemic involvement. It primarily affects posterior teeth, leads to a lateral open bite, and teeth fail to respond to orthodontic forces. MFE is a rare condition similar in presentation to PFE but typically affects only a single tooth, with no impact on adjacent teeth [58]. The study highlighted specific diagnostic criteria for differentiating between PFE and MFE, such as the involvement of the first permanent molar, bilateral presentation, and concurrent dental anomalies. Radiographic findings and genetic tests for mutations in the PTH1R gene were also considered for diagnosis [58,60,61]. The management of PFE was found to be challenging, with treatment options limited. Some cases were managed by accepting the position of the affected teeth, while others required surgical extraction followed by prosthetic replacement or segmental osteotomy with a bone graft. Orthodontic extrusion was generally avoided due to the risk of localized ankyloses. On the other hand, MFE cases were more successfully treated with the extraction of the affected tooth or orthodontic alignment if necessary. The study concluded that an accurate diagnosis between PFE and MFE is essential for appropriate treatment planning. However, due to the rarity of these conditions, a definitive diagnosis might only be made retrospectively, once the patient is dentally mature [58].

**Figure 5:**
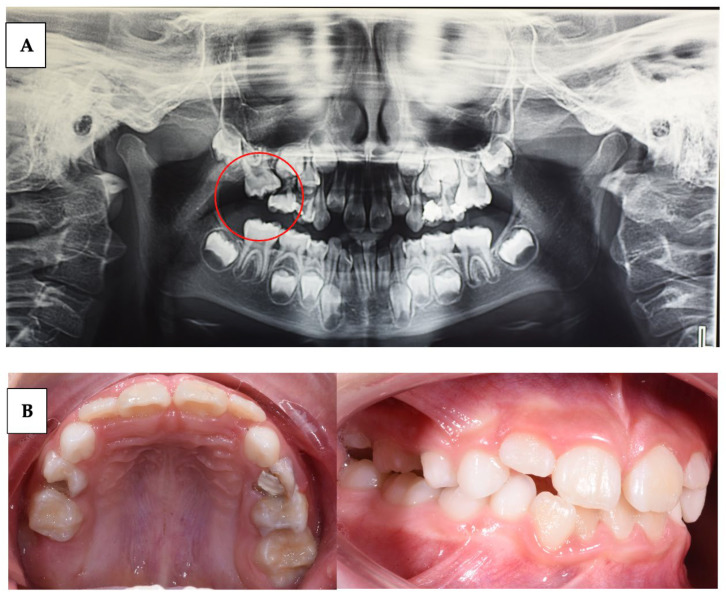
(**A**) Panoramic radiograph of a suspected case of PFE on tooth 16; (**B**) intraoral aspect of suspected PFE on tooth 16.

Rhoads et al. aimed to establish definitive clinical diagnostic criteria to distinguish PFE from other eruptive disorders, particularly ankylosis, a condition in which cement fuses with bone [57]. The researchers collected data from patients diagnosed with PFE, either through genetic analysis confirming the mutation in the PTH1R gene or based on medical records alone. They also included a group of patients diagnosed with ankylosis based on clinical criteria. By comparing the clinical characteristics of the genetic PFE cohort with those of the clinical PFE and clinical ankylosis cohorts, the study aimed to identify the distinctive features specific to PFE. Genetic analysis revealed that PFE patients with confirmed mutations in the PTH1R gene showed some consistent clinical features. In addition, the study discussed the association of PFE with concomitant dental abnormalities and Class III malocclusion suggesting a possible genetic connection between dental and skeletal disorders given the high prevalence of Class III patterns in patients with PFE (Figure 5) [57].

### 3.2. Correlation of PFE and PTH1R

The identification of afflicted people who frequently exhibit hypodontia and have a family history of complications with tooth eruption implies a genetic component in the genesis of PFE. This has been linked to heterozygous mutations in the PTH1R gene on chromosome 3p21.31 (OMIM 168468), which encodes a receptor for PTH [52]. PTH is a key player in calcium homeostasis. When calcium levels in the blood drop, PTH is released to increase calcium absorption in the intestines and release calcium from bone storage, ultimately maintaining blood calcium levels [33]. This hormonal regulation is vital during tooth eruption because calcium is one of the essential minerals required for the formation and mineralization of tooth structure, primarily dentin and enamel [62]. It contributes to the structural integrity of teeth, the health of the surrounding jawbone, muscle movements involved in eruption, nerve function, and the regulation of calcium levels in the body. Any disturbance in calcium metabolism, including dysregulation of PTH, can potentially hinder proper tooth eruption and lead to dental pathologies [63].

Exome analysis identified a novel missense mutation in the CLPP gene in a consanguineous Saudi family expanding the clinical spectrum of Perrault Syndrome type 3 [14].

Using the WES method, Jelani et al. describe here a unique pathogenic alteration of PTH1R (p.Val204Glu) in a Saudi family suffering from PFE. In comparison to the dominant changes linked with PFE to date, the recessive change found in the PTH1R transmembrane domain creates a more severe phenotype. The intrafamilial phenotypic heterogeneity in our cases broadens the range of genotype–phenotype association, even though recessive and dominant mutations of PTH1R have already been documented for overlapping symptoms affecting both bone and dentition. Additionally, in such family instances, the screening for PTH1R changes may be helpful in the therapeutic management of PFE patients [52].

In their study, Le Norcy et al. discovered that most patients (83%) had inappropriate dental eruptions, notably a failure of the primary molars to erupt, which resulted in severe infraocclusion. PFE associated with the g4, heterozygous PTH1R polymorphisms is characterized by an incomplete or complete eruption of non-enclosing primary teeth. The growing teeth of human foetuses with Blomstrand chondrodysplasia and biallelic mutations in PTH1R were gradually impacted by the surrounding bone, despite the fact that they had normal bud development, a normal tooth count, and the proper histological layers [55].

PFE is associated with autosomal dominant mutations in PTH1R; there have been no instances of PFE patients with this mutation presenting with additional skeletal issues, even though the diagnosis of PFE has a poor dental prognosis [64].

Frazier-Bowers et al. performed a mutational investigation of the PTH1R gene based on polymerase chain reaction to ascertain the genetic contribution of PTH1R in 10 families with PFE. Two new autosomal dominant mutations in PTH1R (c.996_997insC and C.572delA), which occur in the coding area and result in a shortened protein, were discovered by sequence analysis of the PTH1R gene’s coding sections and intron-exon boundaries in 10 families (n = 54) and 7 isolated individuals. In eight of the ten families with PFE, no functional (nonsynonymous) changes in PTH1R were found. In addition, four of the families and one isolated instance exhibited synonymous single nucleotide polymorphisms. PTH1R mutations were found in five PFE patients from two families who also had osteoarthritis. They suggest that autosomal dominant PTH1R mutations that cause PFE may also be linked to arthritis. In the absence of any known skeletal system symptoms, a dose-related model can account for isolated PFE and osteoarthritis [51].

According to Grippaudo et al.’s research, mutations of the PTH1R gene are to blame for the eruption’s failure, which has a variety of clinical manifestations that are compatible with PFE descriptions. Early in the deciduous dentition and later in the permanent dentition, several phenotypes can be observed [10]. In addition to ensuring an early diagnosis, a clinical diagnosis along with genetic testing for the PTH1R gene will also serve as a signal to screen prospective afflicted parents.

The study by Pilz et al. [22] involved 36 patients with tooth eruption abnormalities in the posterior jaw segments. Of these patients, 23 were identified as carrying mutations in the PTHR1 gene, while the other 13 showed no known mutations in the gene. The authors sought to distinguish between patients with mutations in the PTHR1 gene and those without such mutations on the basis of clinical and radiographic criteria, concluding that these may be useful in formulating a preliminary opinion about the presence of PTHR1 gene dysfunction in a patient. They emphasize, however, the importance of performing human genetic analysis when an abnormality in the PTHR1 gene is suspected, as some eruption abnormalities may not be related to mutations in the PTHR1 gene [22].

Reis et al. [56] explored the topic of PFE. Previous studies have shown that PFE is caused by heterozygous mutations in the G protein-coupled receptor gene for parathormone (PTH) and parathormone-related protein (PTHrP). Because PTHrP shares the same receptor and Gsα-coupled, PTH-like protein, the hypothesis was put forward that resistance to PTHrP might also be present in PFE, thus contributing to phenomena such as teething failure and other dental manifestations. In the present study, researchers measured plasma PTHrP levels and investigated the relationship between PFE and dental manifestations in 19 patients with Pseudohypoparathyroidism (PHP). However, they found no significant association between plasma PTHrP levels and dental manifestations of PFE or other manifestations of PHP. Thus, the possibility of local resistance to PTHrP in the enamel epithelium and chondrocytes was suggested, which is responsible for the different effects on tooth formation than systemic PTHrP levels [56,65].

Kanno et al. investigated a genetic mutation in a gene associated with PFE. The mutation was found in six individuals across two generations and demonstrated an autosomal dominant inheritance pattern [53]. The mutation was predicted to affect the splicing processes of the PTH1R pre-mRNA, likely leading to protein truncation. The PTH1R gene encodes a family B G-protein-coupled receptor that plays a role in various biological functions when activated by PTH or parathyroid hormone-related protein [18]. Despite the same genetic mutation, the severity and asymmetry of the PFE phenotype varied significantly [53]. The researchers suggested that regional and temporal factors might contribute to the pathogenesis of the condition. Tooth eruption appeared to be influenced by microenvironmental factors, epigenetics, and local influences. Interestingly, enamel and dentin were not affected in individuals with this genetic mutation, and dental anomalies were not detectable with clinical and radiographic methods [53,66]. The study proposed that the onset of PFE may occur during puberty but becomes more evident in adulthood. Some cases of PFE were similar to secondary retention, characterized by eruption impairment after tooth emergence. Ankylosis was ruled out as an etiological factor, as affected teeth responded to orthodontic traction before becoming ankylosed. However, small points of ankylosis may exist in affected teeth. The severity of open bite in molars could be attributed to a higher prevalence of affected interradicular surfaces [53].

The study also reported a case of cervical root resorption associated with PFE, which may suggest a possible link between PTH1R gene mutations and this condition. Overall, the study concluded that PFE is closely related to disturbances in periodontal ligament metabolism, leading to genetically determined ankylosis and resulting in variable clinical presentations [53].

Grippaudo et al. presented the genetic analysis of patients with Familial Pseudo-Partial Eruption (PFE), a rare dental disorder affecting tooth eruption. A total of 44 patients and 3 mothers were included in the study, of which 30 were identified as carriers of variants of the PTH1R gene associated with PFE [10]. The remaining 17 patients did not show any variants. Gender differences were not observed among the variant carriers. Clinical and radiographic criteria for PFE were identified and found in almost all patients with pathogenic PTH1R variants, suggesting their potential diagnostic significance. However, a significant percentage (36%) of PFE patients did not carry PTH1R variants, indicating the involvement of other genes in tooth morphogenesis and eruption [10]. Notably, Parathyroid Hormone-related Peptide (PTHrP) emerged as a potential candidate, warranting further genetic studies. The study highlights the importance of genetic testing in confirming PFE diagnoses and recommends cautious orthodontic treatment in patients with a high probability of PFE [10].

The aim of Stellzig-Eisenhauer A. et al.’s study was to investigate the genetic basis of non-syndromic PFE in families where at least two members were affected [19]. The study followed an interdisciplinary approach, combining clinical and molecular genetics methods. Inclusion criteria for the clinical diagnosis of non-syndromic PFE were defined, and OPTs of all patients were used for radiological diagnosis [19]. Genome-wide coupling analysis and direct DNA sequencing of candidate genes were performed on affected and unaffected individuals. The study identified the gene responsible for non-syndromic PFE, which is the PTHR1 gene; heterozygous mutations in this gene were found in affected patients [19].

### 3.3. PFE Treatment

The management of PFE, a rare genetic abnormality affecting tooth eruption, presents a complex challenge for dental professionals. Traditional surgical and orthodontic interventions have proven ineffective, but after the patient’s growth is complete, a range of multidisciplinary treatment strategies can be considered to address the characteristic malocclusion associated with PFE.

The study of la Monaca et al. [54] reviews the frequency and treatment of eruption failure of permanent first and second molars. This condition is relatively rare but has significant clinical importance because molars play a key role in proper dentoskeletal development. The study showed that early treatment, particularly in younger patients with an incomplete root development stage, led to positive results in most cases. Several treatment modalities were used, including orthodontic uprighting, molar extraction, orthodontic surgery and surgical resection, each with varying results depending on individual patient characteristics and the degree of eruption of the affected molars [54]. Surgical-orthodontic uprighting was the most successful treatment, followed by surgical exposure, orthodontic uprighting, and surgical repositioning. Third molar extraction alone for second molar retention showed a relatively good success rate. Factors such as early diagnosis and coverage of osteomucosal or mucosal crowns were associated with standbetter treatment outcomes [54].

In mild cases, dental restoration with onlays and dental crowns can help. However, it is essential to avoid permanent restorations until the vertical jaw growth is complete. For moderately severe cases, extraction of affected teeth followed by dental implants may be a viable option, with bone grafts if necessary [67,68,69,70].

In severe cases with significant loss of alveolar bone height, a removable prosthesis might be the most appropriate choice [50]. Orthodontic and orthopedic approaches are also utilized for treating PFE. Orthodontic treatment is often needed to align teeth and correct malocclusion, but it is usually planned after growth is complete since early orthodontics is not effective in modifying dento-alveolar growth in PFE [50]. In more severe cases, an orthodontic approach involving orthopedic devices like face masks or functional appliances may be necessary to influence jawbone growth and address the malocclusion. Customized treatment planning according to the severity of PFE is of utmost importance. Each patient’s condition varies, making it crucial to develop personalized treatment plans based on accurate assessments, including clinical evaluations, panoramic X-rays, and, if needed, genetic testing to confirm mutations in the PTH1R gene. The involvement of experienced specialists in managing PFE patients is vital for successful treatment [50,70,71,72].

The article by Wagner et al. emphasizes the Interdisciplinary approach involving oral biologists, pediatric dentists, orthodontists, and oral surgeons, working together in multidisciplinary teams. However, the lack of a unanimous consensus on treatment choices and timing makes patient management challenging. Traction of partially or non-erupted teeth requires careful evaluation by the multidisciplinary team, considering the possibility of traction failure and potential alterations to the occlusal plane [59]. Caries and other complications can occur due to gum continuity interruptions and difficult access during treatment. In cases of partial eruption, overlays can be used for improved function and occlusal stability. The management of deeply impacted teeth presents challenges due to poor visibility and accessibility, sometimes necessitating surgical extraction or retention under a removable denture. Complex implant rehabilitation might be required after avulsion, often involving bone augmentation procedures like autologous bone grafts [59].

Customised treatment planning tailored to each patient’s unique clinical situation, age, and growth is essential for effective PFE management. Genetic diagnosis through sequencing of the PTH1R gene can play a crucial role in preventing ineffective orthodontic traction, managing complications, enabling early PFE diagnosis, and guiding the treatment of offspring with the condition. As research continues, a collaborative and personalized approach will help improve outcomes and enhance the quality of life for PFE patients.

## 4. Limitations

While the discussions and research in the text provide valuable insights into the diagnosis, treatment, and genetic basis of PFE, there are certain limitations that need to be acknowledged. Firstly, the small number of studies involved. Furthermore, the studies cited in the text might have been conducted on a relatively small sample size due to the rarity of PFE, which could limit the generalizability of the findings. Moreover, the text does not explore potential confounding variables or the impact of environmental factors on PFE development, which could play a role in the clinical manifestations of the condition. Furthermore, the genetic analysis of PFE is still an ongoing area of research, and there might be other genetic factors involved in tooth eruption abnormalities that have not been fully explored in the text. Finally, the text does not address the potential ethical implications of genetic testing for PFE and the importance of informed consent and genetic counselling for affected individuals and their families. Despite these limitations, the text serves as a valuable overview of the current knowledge on PFE, but further research is warranted to gain a comprehensive understanding of this rare dental disorder. Conclusions

In conclusion, the diagnosis and management of PFE and MFE have presented a significant challenge within the field of dentistry. These conditions, though rare, have a profound impact on oral health and the overall well-being of affected individuals.

One of the primary complexities in addressing these disorders lies in their infrequency, often requiring retrospective diagnoses. Managing PFE has revealed itself as a complex undertaking, characterized by a spectrum of treatment modalities that must be meticulously tailored to the unique characteristics and severity of each case. Multidisciplinary collaboration and genetic testing have arisen as indispensable tools in effectively addressing these disorders.

Genetic factors have emerged as fundamental in comprehending the etiology of these conditions. Mutations within genes such as PTH1R and PTHR1, associated with PTH and calcium metabolism, have been strongly implicated in the pathogenesis of PFE. While genetic testing holds promise as a means of early diagnosis and personalized treatment, it is important to acknowledge the potential genetic heterogeneity and involvement of other genes in contributing to these conditions.

Treatment focuses on identifying and managing the underlying causes. In some cases, it is possible to resort to conservative therapies such as the use of separators or orthodontic devices aimed at correcting the position of the teeth. However, in more complex cases, it may be necessary to resort to surgical interventions, such as surgical exposure of the tooth through specific procedures. It is essential that the treatment is personalized according to the specific needs of the patient, considering his age, the severity of the defect and other related factors. Careful evaluation by qualified professionals is essential to ensure the best possible outcome and preserve long-term dental health.

The multifaceted and diverse nature of these disorders underscores the critical importance of continued research, interdisciplinary cooperation, and an individualized approach to PFE management. This approach encompasses early diagnosis when feasible and a meticulous evaluation of the condition’s severity, leading to the formulation of tailored treatment plans. Such an approach not only serves the best interests of the individuals affected but also advances our comprehension of the genetic underpinnings of tooth eruption disorders. As research advances, it fosters optimism for improved outcomes and an enhanced quality of life for those confronted with PFE and its related conditions.

Further research is needed to explore the complex genetic basis of PFE fully and improve treatment outcomes for affected individuals.

## Figures and Tables

**Table 1:** Physiological times of teeth eruption [38].

Lower first molars	6–7 years
Upper first molars	6–7 years
Lower central incisors	6–7 years
Upper central incisors	7–8 years
Lateral lower incisors	7–8 years
Upper lateral incisors	8–9 years
Upper canines	9–10 years
Inferior canines	9–10 years
Inferior premolars	9–10 years
Upper premolars	10–12 years
Lower second molars	11–13 years
Upper second molars	11–13 years
Third molars	17–25 years

**Table 2:** Descriptive summary of item selection.

Author (Year)	Study Design	Number of Patients	Aim of the Study	Material and Methods	Results
Aziz et al. (2019) [50]	Case series	3	To mark a PFE family with mutation of the PTH1R genus in primary and permanent dentition.	Clinical examination, radiological examination, and molecular DNA testing.	Once growth is complete, various multidisciplinary treatment strategies can partially resolve the posterior open bite malocclusion that is characteristic of this disorder.
Frazier- Bowers S.A. et al. (2014) [51]	RCT	61	Familial PFE genetic variants can be inherited in an insufficiently penetrant way and may be linked to an early start of osteoarthritis.	Polymerase chain reaction-based mutational analysis confirmed PTH1R’s genetic role in 10 families with PFE. Two new autosomal dominant variants (c.996_997insC and C.572delA) were discovered in ten families and seven solitary individuals.	PTH1R autosomal dominant mutations cause PFE and osteoarthritis; a dose-dependent model may explain isolated osteoarthritis and PFE without known skeletal system symptoms.
Grippaudo C. et al. (2018) [10]	RCT	51	To investigate the possibility that both primary and permanent teeth may be affected by mutations in the parathyroid hormone 1 receptor (PTH1R).	A study of 29 individuals with infraoccluded teeth involved saliva samples, DNA retrieval, and sequencing of PTH1R gene coding regions. Mutations were examined for genetic information.	The PTH1R gene contains novel mutations affecting primary molars affected by PFE, paving the way for early genetic diagnostics and appropriate care.
Grippaudo et al. (2021) [3]	RCT	44	To interrogate and validate the correlation between clinical features and the presence of PTH1R variants in a cohort of patients with suspected PFE and their family members.	Clinical and genetic analysis (PTH1R sequencing).	Patients with pathogenic variants of the PTH1R gene exhibit clinical traits of PFE. Genetic testing identifies PTH1R variants in about 64% of PFE patients.
Jelani M. et al. (2016) [52]	Case Series	6	Utilizing whole-exome sequencing (WES) analysis, determine the genetic aetiology of the non-syndromic main failure of dental eruption in a five-generation related Saudi family.	Researchers performed WES on all four afflicted family members using the 51 Mb SureSelect V4 library kit, followed by Illumina HiSeq2000 sequencing.	The results demonstrate the usefulness of WES as a molecular diagnostics tool for this uncommon condition and extend the clinical range of PTH1R pathogenicity.
Kanno et al. (2017) [53]	Case series	18	Determine the genetic and clinical characteristics of a family with 11 PFE-affected members after 20 years of observation.	Clinical and genetic analysis.	PFE disrupts periodontal ligament metabolism, increasing genetically determined ankylosis. Severe open bites occur earlier, and affected teeth maintain eruptive potential even in adulthood.
La Monaca et al. (2019) [54]	Retrospective study	125	The objective of this study was to examine information from patients experiencing either unsuccessful or delayed eruption of their first and second permanent molars, with the goal of evaluating the efficacy of the treatment approaches employed.	The authors classified eruption disorders based on root development, using terms like “retention” and “inclusion” to describe molars before and after apical root closure. They also used “early diagnosed condition” to describe anatomical, topographical, or pathological conditions that could interfere with eruption processes.	The article highlights that treatment outcomes are most favourable for younger patients with undeveloped molar roots, with early diagnosis and prompt action significantly impacting effectiveness. Surgery uprighting has the highest success rate, while surgical-orthodontic uprighting has a lower success rate.
Le Norcy E. et al. (2020) [55]	RCT	19	Researchers investigated dental, oral, and craniofacial characteristics in offspring with iPPSD2 and maternal GNAS inactivating mutations to determine malformation prevalence and specificity.	To determine the prevalence and specificity of the defects, they performed a thorough examination of dental and craniofacial characteristics in 19 individuals with iPPSD2 and maternal GNAS inactivating mutations.	Patients with parental GNAS mutations and iPPSD2 developed particular dental and craniofacial malformations.
Pilz et al. (2014) [22]	Retrospective study	36	This study’s goal was to determine whether it is possible to distinguish between PTHR1-mutation carriers and noncarriers based on radiological and clinical findings.	This study found that posterior teeth are more frequently affected, with eruption disturbances, resorption of alveolar bone coronal, involvement of both deciduous and permanent teeth, impaired vertical alveolar-process growth, and severe posterior open bite findings.	It Is possible to significantly improve the specificity of selecting non-affected patients from among suspected instances of PFE associated with PTHR1 gene mutations by carefully assessing clinical and radiographic parameters.
Reis et al. (2016) [56]	Cross sectional study	19	To investigate patients with PTH resistance and molecular diagnosis of PHP and evaluate dental and radiological manifestations associated with the condition.	Genetic tests were conducted on GNAS and STX16 genes linked to PHP syndromes. Biochemical analyses measured serum calcium, phosphate, PTH, and PTHrP levels. Radiological and dental evaluations were performed using DPR to detect FTE and dental anomalies.	Identified mutations and methylation defects in the GNAS gene associated with PTH in a group of patients. Radiological and dental evaluation detected the presence of incomplete tooth eruption and other dental anomalies in patients with the condition.
Rhoads et al. (2013) [57]	Retrospective study	64	Define the clinical diagnostic criteria that separate PFE from other eruption disorders, particularly ankylosis, by utilizing a special dataset.	PFE is diagnosed by a PTH1R gene mutation, with clinical criteria including permanent first molar involvement, supracrestal presentation, eruption path obstruction, second premolar involvement, multiple adjacent teeth, bilateral presentation, Class III malocclusion, and dental abnormalities.	PFE is a condition affecting first molars, adjacent teeth, and adjacent teeth. It can cause infraoccluded second premolars and bilateral affection. In absence of trauma, treatment history, genetic information, or periodontal ligament obliteration, PFE and ankylosis may be clinically indistinguishable.
Sharma et al. (2016) [58]	Retrospective descriptive study	29 (15 as PFE and 14 as MFE)	Identify management techniques and confirm diagnostic criteria that will help differentiate between PFE MFE of permanent molars.	PFE affects the first molar and can cause unerupted or partially erupted teeth, causing open bites and a clear eruption pathway in patients. It can affect distal teeth, primary dentition, and multiple teeth.	The authors suggest a protocol in the form of a flow diagram based on the findings of the study and current protocols to help with the appropriate diagnosis and management of PFE and MFE.
Stellzig-Eisenhauer et al. (2010) [19]	Case series	15	Identify a genetically verified diagnosis of PFE to improve treatment choice.	Clinical and Molecular Genetics Analysis	Molecular genetic analysis of the PTHR1 gene revealed three distinct heterozygous mutations. Unaffected patients exhibited no mutations.
Wagner et al. (2023) [59]	Case series	3	Describe the variability of clinical presentations of PFE associated with pathogenic variants of PTHR1	Clinical examination, radiological examination, and molecular DNA testing	Multidisciplinary complex treatment, from child to adulthood.

## Data Availability

Not applicable.

## Abbreviations

PFE	Primary Failure Eruption
MFE	Mechanical Failure of Eruption
OPT	Orthopantomograph
PHP	Pseudohypoparathyroidism
PTH	Parathyroid hormone
PTHR1	Parathyroid hormone 1 receptor
PTHrP	Parathormone-related protein
PTHrP	Parathyroid Hormone related Peptide
WSE	Whole-exome sequencing
